# Prevalence of *Helicobacter pylori *in HIV-infected, HAART-naïve Ugandan children: a hospital-based survey

**DOI:** 10.1186/1758-2652-14-34

**Published:** 2011-06-30

**Authors:** Elin Hestvik, Thorkild Tylleskar, Grace Ndeezi, Lena Grahnquist, Edda Olafsdottir, James K Tumwine, Deogratias H Kaddu-Mulindwa

**Affiliations:** 1 Centre for International Health, University of Bergen, Årstadveien 21, N-5009 Bergen, Norway; 2 Department of Paediatrics, Haukeland University Hospital, N-5021 Bergen, Norway; 3 Department of Paediatrics and Child Health, Makerere University School of Medicine, College of Health Sciences, PO Box 7072, Kampala, Uganda; 4 Department of Microbiology, Makerere University Medical School, PO Box 7072, Kampala, Uganda; 5 Department of Women's and Children's Health, Karolinska Institute, 17176 Stockholm, Sweden

## Abstract

**Background:**

The aim of this survey was to determine the prevalence of and factors associated with *Helicobacter pylori *(*H. pylori*) colonization in HIV-infected, highly active antiretroviral therapy-naïve Ugandan children aged 0-12 years.

**Methods:**

In a hospital-based survey, 236 HIV-infected children were tested for *H. pylori *colonization using a faecal antigen test. A standardized interview with socio-demographic information and medical history was used to assess risk factors. A cluster of differentiation 4 (CD4) cell percentage was prevalent in most children.

**Results:**

The overall prevalence of *H. pylori *in the HIV-infected children was 22.5%. Age-specific prevalence was as follows: up to one year, 14.7%; 1-3 years, 30.9%; and 3-12 years, 20.7%. HIV-infected children who were more seriously affected by their disease (low CD4 cell percentage or WHO clinical stage II-IV) were less likely to be colonized with *H. pylori*. There was a trend for a lower prevalence of *H. pylori *in children who had taken antibiotics for the preceding two weeks (21.6%) than in those who had not taken antibiotics (35.7%). There was no statistically significant difference in prevalence by gender, housing, congested living, education of the female caretaker, drinking water or toilet facilities.

**Conclusions:**

HIV-infected, HAART-naïve Ugandan children had a lower prevalence of *H. pylori *colonization compared with apparently healthy Ugandan children (44.3%). Children with a low CD4 cell percentage and an advanced clinical stage of HIV had an even lower risk of *H. pylori *colonization. Treatment with antibiotics due to co-morbidity with infectious diseases is a possible explanation for the relatively low prevalence.

## Background

Sub-Saharan Africa accounts for 67% of all people living HIV, and carries the highest burden of the global HIV epidemic [1]. In Uganda, it has been estimated that 1.1 million people, including 120,000 children, were living with HIV in 2008 [2]. The gastrointestinal tract is the largest immunological site of the body and HIV infection profoundly impacts on gut function [3,4]. HIV-infected children are affected by numerous gastrointestinal problems [5].

*Helicobacter pylori*, which can cause chronic gastritis, is associated with recurrent peptic ulcers and gastric cancer [6,7]. It is one of the most common causes of bacterial infection in man [8,9]. It was first isolated and cultured from the antrum of patients with gastritis by Warren and Marshall in 1983 [10]. *H. pylori *colonization is thought to be acquired early in life. Early colonization in children living in poor socio-economic conditions has been demonstrated, and several studies have shown a high prevalence of *H. pylori *among people in low-income countries [11-15]. The overall prevalence was 44.3% in our recently published study on apparently healthy, urban Ugandan children [15].

Published data on *H. pylori *infections in HIV-infected persons are mainly based on adults [16] and are from non-epidemic areas [17], and many studies have undertaken serological tests [18] or have been conducted on persons referred for gastrointestinal complaints [19]. These studies report diverging estimates of the prevalence of *H. pylori *[20]. To the best of our knowledge, there seem to be no studies on the prevalence of *H. pylori *in HIV-infected children living in sub-Saharan Africa. In a study designed to describe the findings in HIV-infected South African children who underwent gastroscopy, rates of *H. pylori *colonization were reported, and only one out of 26 children was colonized [21].

There are currently four distinct methodologies for *H. pylori *detection and/or identification: (1) ^13^Urea breath test [22,23]; (2) gastroscopy with biopsies and culture; (3) serology tests; and (4) antigen tests. The ^13^Urea breath test or invasive methods, such as gastroscopy with biopsies and/or urease tests, used to be the "gold standard" for detection of *H. pylori*. A ^13^Urea breath test is time consuming and personnel dependent. A gastroscopy should be performed when a child presents clinical symptoms for diagnosis and is not justified for mere identification of *H. pylori*. Serological tests are available, but have several drawbacks: (1) they do not discriminate between current and past infections; (2) they show low specificity in children and are thus of little use [24,25]; and (3) no data is available about the specificity and sensitivity of serological tests in HIV-infected individuals with immunodeficiency and altered antibody production.

The faecal monoclonal antigen test has a high sensitivity, specificity and accuracy in children: 91-100, 84-96 and 94-96%, respectively [26-28]. In a review on the incidence of *H. pylori *in HIV-infected patients [20], the use of the faecal antigen test is recommended for further studies due to its higher specificity in this population. It can be used on humans of all age groups, gives a rapid result without being invasive, and is not affected by acid-regulating medicines.

Our main objective was to determine the prevalence and factors associated with *H. pylori *colonization in HIV-infected, highly active antiretroviral therapy- (HAART-) naïve children aged 0-12 years in urban Kampala, Uganda.

## Methods

### Study site and data collection

The survey was conducted from February to October 2008 at the Department of Paediatrics and Child Health, Mulago National Referral Hospital, Kampala, a government-run hospital. It assumes the role of the local hospital for the people living in the area of Mulago Hill and, at the same time, the role of a national referral hospital for Uganda. Participants were enrolled from the general paediatric medical wards, the acute care unit, the ward for malnutrition and the paediatric infectious diseases clinic.

We decided in advance on an enrolment period of nine months. The data was collected by a doctor with experience in data collection and in paediatric HIV pre- and post-test counselling, as well as diagnosis and treatment of paediatric HIV. She was fluent in the local languages and English. GN and EH trained her in stool sampling, interview technique and ethical issues. GN was available for consultation if necessary. Children admitted during the enrolment period to the wards we have mentioned were invited to participate in the study if they were HIV infected, but HAART naïve, aged 0-12 years, and only after receiving informed consent from their caretaker. The ward matrons were asked to identify the eligible children and their caretakers. All those so identified children and caretakers were invited to participate.

The HIV status of the children was known before enrolment from routine testing as part of the medical service at the Department of Paediatrics and Child Health. HIV testing followed the Ugandan national guidelines [29] that closely follow the World Health Organization (WHO) guidelines. Children over 18 months of age were tested using a rapid blood test with a sensitivity rate > 98%. To confirm positive test results, a second test with a different antigenic specificity was used. If there was discordance between the two tests, an ELISA test (tie-breaker) was used to make a final diagnosis. For children under 18 months of age, a polymerase change reaction test was used to give a reliable HIV diagnosis.

### Study population

**Figure 1:**
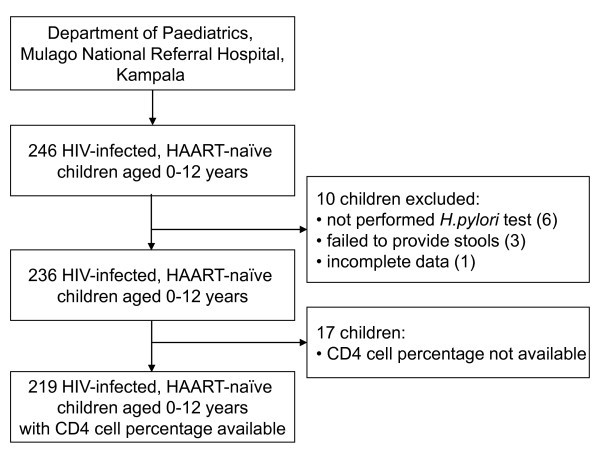
**Study profile**.

The study population (Figure 1) consisted of 246 HIV-infected, HAART-naïve children aged up to 12 years. Only 4.1% of the eligible children (10/246) were not included in the final analysis as a result of: no *H. pylori *test performed (six); failure to produce a stool sample in three days (three); and providing an incomplete questionnaire (one). In 219 of the 236 participants, CD4 cell counts expressed as percentages were available. We classified CD4 cell percentage as high or low with limits defined by age: (1) for children < 12 months, high if CD4 cell percentage was > 25%: (2) for 12-36 months, high if CD4 cell percentage was > 20%; and (3) for ≥36 months, high if CD4 cell percentage was > 15%. The limits chosen were concurrent with those recommended for starting HAART according to the WHO guidelines available at the time of the study [30]. All children were clinically categorized using the WHO staging system for HIV-infected children [31] since it is recommended for evaluating the need to start up HAART in children when CD4 cell counts are unavailable [30].

### *H. pylori *stool antigen test

Stool samples requested from each participating child were collected in airtight containers at the time of the encounter, the end of the day, or the following morning. They were transported from the ward to the laboratory twice daily and stored in a +4°C fridge for a maximum of 24 hours before analysis by the *H. pylori *stool antigen test, HpSA^®^ImmunoCardSTAT, as per the manufacturer's instructions. A standard positive control test was performed after every 20 tests, all of them being verified as positive. The HpSA^®^ImmunoCardSTAT is a rapid lateral-flow immunoassay that utilizes a monoclonal anti-*H. pylori *antibody as the capture and detector antibody. Approximately 100 μl of stool was transferred into the sample diluent vial and vortexed for 15 seconds. Four drops of the specimen were applied to the test and the result was read after five minutes. The results were reported as positive or negative based on the manufacturer's cut-off values.

### Statistical analysis

Data from the questionnaires, the results of the faecal antigen test and CD4 cell percentages were doubly entered using EpiData version 3.1 (http://www.epidata.dk). Data quality was ensured through careful selection and training of research assistants, supervision, and field editing by use of the "check" module at data entry combined with double data entry and validation. The "checks" at data entry were limits set by the study team to ensure that it was not possible to enter obviously wrong information. For example, a child can measure only between 48 cm and 180 cm (it is not possible to enter other data), and many answers can only be "yes" or "no".

After entering all data twice in separate files, the two separate data files were validated by comparison and any non-matching data were checked manually against the original paper form. The data were exported to SPSS version 17.0 for statistical analysis. To explore the prevalence of *H. pylori *and its association with other factors, bivariate logistic regression and multiple logistic regression were performed. Adjustments were made in the multiple logistic regression analysis for age, sex, CD4 cell percentage, clinical WHO staging, type of housing, number of people in the same household, education of the mother or female caretaker, sources of drinking water, toilet type (pit latrine), sharing of the toilet with other families, reported abdominal pain, wealth index, and drugs taken.

Due to the lack of known CD4 cell percentages in 17 participants, the multiple logistic regression analysis involved only 219 participants. The confidence interval (CI) reported was set at 95%, and the significance level was set at 0.05. To explore the socio-economic status of the participants, principal component analysis (PCA) was used. Twelve questions encompassed socio-economic status (composed of assets in the household, sources of power available for the family, standard of housing for the child, and if the family were farmers or owned their own land and/or house), and we carried out PCA for these questions. The model captured ~79% of our results; the Kaiser-Meyer-Oklin value was 0.79, exceeding the recommended value of 0.6, and the Barletts Test of Sphericity reached statistical significance. PCA revealed the presence of three components with Eigen values exceeding 1. The first principal component was used as our wealth index, explaining 30.5% of the variance. The wealth index was ranked and categorized into three tertiles (1 - poorest, 2 - poorer, 3 - least poor) that were equally distributed.

### Ethics

Ethical approval was obtained from Makerere University, Faculty of Medicine, Research and Ethics Committee in Uganda, and the Regional Committee for Medical and Health Research Ethics, West-Norway (REK-VEST) in Norway. The data collectors were trained in ethical issues prior to the survey. Oral and written information about the study was given to the caretakers either in English or the local language. Informed consent was obtained from all the caretakers of the participants in the study. If the doctor found the medical history of a participating child suspect of gastritis and the child tested positive for *H. pylori*, the child was given triple therapy of amoxicillin/claritromycin/omeprazole for one week. All children participating in the study were independently managed for their medical needs by the doctor in charge of the ward.

## Results

The mean age (± SD) of the 236 participants who completed the study was 2.9 (2.8) years; for girls 2.8 (2.8) years and boys 3.1 (2.8) years. The youngest enrolled child was 1.5 months. There were 19 children younger than six months enrolled. The genders were equally represented in the survey: 121 (51.3%) girls and 115 (48.7%) boys.

The overall prevalence of *H. pylori *antigen in the 236 children was 22.5 % (Table 1).

**Table 1:** Prevalence of *Helicobacter pylori *in Ugandan HIV-infected children by age groups

Age categories	Total number N	*H. pylori *positiv*e *n	*H. pylori *prevalence % (95% CI)
0 < 1 year	68	10	14.7 (6-23)
1 < 3 years	81	25	30.9 (21-41)
3 < 6 years	53	12	22.6 (11-34)
6 < 9 years	22	4	18.2 (1-36)
9 < 12 years	12	2	16.7 (8-41)
Total	236	53	22.5 (17-28)

Age-specific prevalence was: (1) for up to one year, 14.7%: (2) for 1-3 years, 30.9%; and (3) for 3-12 years, 20.7%. The difference in prevalence between the youngest children and the group aged 1-3 years was significant, also after adjusting for the other factors in the multiple logistic regression analysis (Table 2). The lower prevalence after the age of three years was not statistically significant. There was no difference in colonization rates of *H. pylori *by gender.

**Table 2:** *Helicobacter pylori *colonization in HIV infected, treatment naïve children and associated factors

	Number N	HP positive n (%)	Unadjusted odds ratio (95% CI)	p value	Adjusted odds ratio^1 ^(95% CI)	p value
**Age groups**						
0 < 1 year	68	10 (14.7)	1		1	
1 < 3 years	81	25 (30.9)	2.6 (1.1-5.9)	0.02	2.8 (1.1-6.8)	0.03
3 < 12 years	87	18 (20.7)	1.5 (0.6-3.5)	0.34	1.2 (0.5-3.2)	0.65
						
**Sex**						
Male	115	22 (19.1)	1		-	
Female	121	31 (25.6)	1.5 (0.8-2.7)	0.23		
						
**CD4 cell percentage**^2^						
High	115	35 (30.4)	1		1	
Low	104	13 (12.5)	0.3 (0.2-0.7)	0.002	0.3 (0.1-0.6)	0.001
						
**Who classification**						
WHO stage I	24	9 (37.5)	1		-	
WHO stage II-VI	212	44 (20.8)	0.4 (0.2-1.1)	0.07		
						
**Type of housing**						
Permanent house	105	23 (21.9)	1		-	
Semi-permanent house	131	30 (22.9)	1.1 (0.6-2.0)	0.86		
						
**Number of people** **in same household**						
2-4	126	28 (22.2)	1		-	
≥5	110	25 (22.7)	1.0 (0.6-1.9)	0.93		
						
**Education of the** **mother/female caretaker**						
Completed primary	83	21 (25.3)	1		-	
school or higher						
Incomplete primary school	153	32 (20.9)	0.8 (0.4-1.5)	0.44		
						
**Drinking water**						
Public tap	138	28 (20.3)	1		-	
Unprotected sources	98	25 (25.5)	1.4 (0.7-2.5)	0.34		
						
**Type of toilet**						
Open pit/pit latrine	228	52 (22.8)	1		-	
VIP latrine/flush toilet	8	1 (12.5)	0.5 (0.1-4.0)	0.50		
						
**Sharing of toilet** **with other families**						
No	72	16 (22.2)	1		-	
Yes	164	37 (22.6)	1.0 (0.5-2.0)	0.95		
						
**Taken drugs last 3 months**^4^						
No	40	10 (25.0)	1		-	
Yes	196	43 (21.9)	0.8 (0.4-1.9)	0.67		
						
**Taken any antibiotics**^3^ **last 3 months**						
No	61	17 (27.9)	1		-	
Yes	175	36 (20.6)	0.7 (0.3-1.3)	0.24		
						
**Taken any antibiotics** **last 2 weeks**						
No	14	5 (35.7)	1		-	
Yes	222	48 (21.6)	0.5 (0.2-1.6)	0.23		
						
**Taken deworming** **medicine last 6 months**						
No	125	23 (18.4)	1		-	
Yes	111	30 (27.0)	1.6 (0.9-3.0)	0.12		
						
**Wealth index**						
Least poor	78	12 (15.4)	1		-	
Poorer	80	19 (23.8)	1.7 (0.8-3.8)	0.19		
Poorest	77	22 (28.6)	2.2 (1.0-4.8)	0.05		
						
**Reporting abdominal pain** **more than 3 times/week**						
No	224	49 (21.9)	1		-	
Yes	12	4 (33.3)	1.8 (0.5-6.2)	0.36		

^1^Adjusted for the 219 participants for whom CD4 percentage were available; adjustment was made for all categories included in the table.

^2^CD4 cell percentage was available for 219 of the 236 participants

^3^Last 2 weeks not included in this category

CD4 cell percentages were available for 219 participants. A low CD4 cell percentage was significantly associated with a lower *H. pylori *colonization rate, with an odds ratio (OR) and 95% confidence interval (OR ± 95% CI) of 0.33 (0.2-0.7), (Table 2). Participants with WHO stage II-VI had lower *H. pylori *colonization (20.8%) compared with those with WHO stage I (37.5%) (Table 3). The difference was not statistically significant (OR = 0.4; 95% CI 0.2-1.1) (Table 2).

**Table 3:** Prevalence of *Helicobacter pylori *in Ugandan HIV-infected children by WHO stage

WHO stage	Total number N	*H. pylori *positive n	*H. pylori *prevalence % (95% CI)
Stage I	24	9	37.5 (17-58)
Stage II	44	8	18.2 (6-30)
Stage III	145	32	22.1 (15-29)
Stage VI	23	4	17.4 (1-34)
Total	236	53	22.5 (17-28)

In the unadjusted analysis, the *H. pylori *colonization was higher among the poorest participants than among the other participants (OR ± 95% CI) of 2.2 (1.0-4.8), but the difference was not statistically significant after adjusting for the other factors in the analysis (Table 2).

There was no statistically significant difference in colonization rate in participants who had taken any kind of drugs or antibiotics within the last three months or two weeks before the survey assessment (Table 2). There was a lower colonization rate in children who had had antibiotics in the last three months (20.6 versus 27.9%) and in the last two weeks (21.6 versus 35.7%), but the difference was not statistically significant. Prophylaxis with cotrimoxazole was common (69.9%), but there was no different in the colonization rate between participants with or without prophylaxis.

There was no statistically significant difference in *H. pylori *prevalence by type of housing, congested living, education of female caretaker, drinking water sources, toilet facilities or reported abdominal pain (Table 2).

## Discussion

In this large survey of HIV-infected children in an African urban setting, we identified a lower colonization rate of *H. pylori *in HIV-infected children compared with healthy children in the same area of Kampala, Uganda [15]. HIV-infected children more seriously affected by their disease (low CD4 cell percentage or WHO stage II-IV) were less likely to be colonized with *H. pylori*.

This is the first survey describing the prevalence of *H. pylori *colonization among HIV-infected, HAART-naïve Ugandan children. This is a novel survey in an epidemic area of HIV with focus on the prevalence of *H. pylori *in HAART-naïve children. Only two previous studies have provided data on prevalence of *H. pylori *in HIV-infected children [32,33], neither of them from endemic areas for HIV. A Belgian study [32] on 23 HIV-infected children of central African ethnic origin and born in Belgium used a serology test to detect *H. pylori *colonization. They found none of the tested children to be colonized compared with 19.2% of children in a control population.

An Italian study [33], using both serology and ^13^Urea breath tests in 45 perinatally HIV-infected children, found a prevalence of 17.7 and 20.0%, respectively. This was not different from a control population, but the HIV-infected and the control patients were both recruited from a socio-economic background predisposing them to *H. pylori *colonization; many of the children had a caretaker involved in intravenous drug abuse [33]. In 26 HIV-infected South African children who underwent gastroscopy, the rates of *H. pylori *colonization were reported, and only one child was colonized [21]. Our survey had a large sample size compared with other studies describing *H. pylori *prevalence in HIV-infected children [21,32,33].

In this survey, we used an active antigen method to investigate the colonization of *H. pylori*. A positive test is evidence of a current infection and not the possibility of a previous infection, which could have been the case had a serological test been used. If a test based on antibody detection was used, a participant with severe immunodeficiency could eventually show a false negative result due to an inadequate immune response. From other studies, we know that the antigen test used has high sensitivity and specificity in non-HIV-infected populations [26,27], and is recommended for screening in HIV-infected population [20].

A weakness is that we have no data on the specificity and sensitivity of this test in HIV-infected, immune-suppressed populations. Another weakness is that the number of children over six years of age was small compared with the rest of the study population, increasing the confidence interval of our estimates for *H. pylori *prevalence in the older age group. We failed to recruit more children over six years of age due to the natural history of AIDS and due to our inclusion criteria being HAART-naïve children.

In the adjusted multiple regression analysis, we had only 219 participants as 7.2% of the study population did not have their CD4 cell percentages measured. This made analysis more complex, but comparison of the models with 219 participants and all 236 participants showed no significant differences in OR with 95% CI or p values. In the analysis in Table 2, some of the factors had a much more skewed distribution and the survey did not have enough power to detect differences in the prevalence of *H. pylori*.

**Figure 2:**
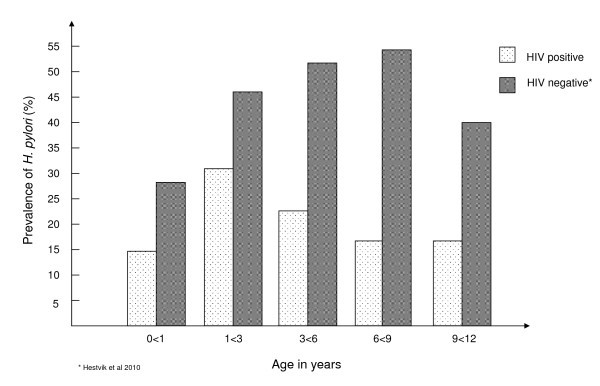
**Comparison of prevalence of *Helicobacter pylori *in apparently healthy and HIV-infected Ugandan children by age**.

We have recently reported the prevalence of *H. pylori *in apparently healthy children in Kampala, Uganda [15]. Apparently healthy Ugandan children had an overall prevalence of *H. pylori *of 44.3%; HIV-infected Ugandan children had an overall prevalence of *H. pylori *of 22.5%. The prevalence in the HIV-infected children was lower in all age groups compared with apparently healthy children (Figure 2).

Although there are limitations, the two studies have similarities. The two study populations have children aged 0-12 years, the gender distribution is similar, the same antigen test was used in both studies, and both studies were performed in urban areas. Sanitation conditions did not differ much between the two populations. The limitations of comparing the two studies are that one group is community based, receiving home visits, and the present study is hospital based, and that in the community-based study, 39% of the participants had taken antibiotics in the last three months versus 74% in the present study.

We identified a statistically significantly lower colonization rate of *H. pylori *in children who had low CD4 cell percentages. A CD4 cell percentage is more accurately used in young children due to the natural decline in the total lymphocyte count and the CD4 cell count [34]. To the best of our knowledge, there are no studies performed in child populations showing differences in prevalence of *H. pylori *according to the CD4 cell count. In adult populations, we find support for our findings: in a study from Argentina [35], the authors concluded that HIV-infected patients with *H. pylori *had a higher mean CD4 cell count than those without *H. pylori*; and a Zambian study [36] showed that HIV-infected adult patients with CD4 cell counts below 200 cells/mm^3 ^were less likely to have positive *H. pylori *serologies (OR 0.29; 95% CI 0.09-0.93).

We found that *H. pylori *colonization was significantly higher in children aged 1-3 years than in children younger than one year of age. This is comparable to data for apparently healthy children from the same region [15], but it has not been described earlier among HIV-infected children in this age group. An Italian study [33] describing colonization of *H. pylori *by age only included three children younger than three years and none younger than one year of age.

We speculate that the colonization rates among the HIV infected are the same as in apparently healthy children, but among the HIV-infected children, accidental eradication is taking place due to the high use of antibiotics in these children; 74% of the children had taken antibiotics within the last three months and 95% of the survey participants were on antibiotics at time of enrolment or had taken antibiotics within the preceding two weeks.

Hospitalization therapy for bacterial infections, worms and protozoa are often given simultaneously if infections are present. These combined therapies can also be effective against *H. pylori *and eradicate it in a proportion of children. The use of antibiotics against opportunistic infections in HIV-infected populations is the most hypothesized explanation for lower colonization rates [19,20,37]. We could only show a trend of lower colonization among children who had used antibiotics. We assume that we could not show a significant difference between those who had used antibiotics and the others due to the large number of participants who had been treated with antibiotics in the past compared with those who had not been treated. The study was not designed to show those differences.

To use a clinical staging system for AIDS is useful and recommended when a CD4 cell count is not available, but it is not recommended for use for initiating HAART if a CD4 cell count is available [30]. We could not demonstrate a significant difference relating to the clinical HIV WHO stage and the prevalence of *H. pylori*, but there was a trend for lower colonization rates in more advanced stages (Table 3). We think this is because many of the criteria used for advanced staging are chronic or recurrent infection. These infections are treated with antibiotics, for example, amoxicillin against upper airway infections; this drug is also recommended as a part of the triple treatment of *H. pylori *[38].

There was no significant difference in prevalence by sex, type of housing, congested living, education of female caretaker, drinking water sources, toilet facilities, reported abdominal pain or wealth index. A possible explanation for the lack of such association, as described in non-HIV-infected children [11,14,15,39], is that the impact of the CD4 cell percentage is very strong and independent of the factors we have mentioned.

## Conclusions

HIV-infected, HAART-naïve, urban Ugandan children had a lower prevalence of *H. pylori *colonization compared with apparently healthy Ugandan children. Children with more advanced HIV (a low CD4 cell percentage and advanced clinical stage of HIV) had lower rates of colonization of *H. pylori*; this might indicate that these children had more frequently been treated with drugs also effective against *H. pylori*. Treatment with antibiotics or other drugs effective against *H. pylori*, due to co-morbidity with infectious diseases, is a likely explanation for the relatively low prevalence.

## Competing interests

The authors declare that they have no competing interests.

## Authors' contributions

EH participated in the conception, design and implementation of the study, statistical analysis, interpretation and writing of the manuscript. TT participated in the conception and design of the study, statistical analysis, interpretation and writing of the manuscript. DKM participated in implementation of the study and performed the HpSA tests. GN participated in design and implementation of the study. LG participated in design of the study, interpretation and writing of the manuscript. EO participated in the conception and design of the study, statistical analysis, interpretation and writing of the manuscript. JKT participated in conception, design and implementation of the study. All authors read and approved the final manuscript.
